# An electronic brachytherapy technique for treating squamous cell carcinoma in situ of the digit: a case report

**DOI:** 10.1186/1756-0500-6-147

**Published:** 2013-04-15

**Authors:** V Elayne Arterbery, Alice C Watson

**Affiliations:** 1 Crittenton Radiation Oncology Center, 1901 Star Batt Drive, Rochester Hills, MI, 48309, USA; 2 Department of Dermatology, University Physician Group-Georgetown, Professional Building, Suite 208, Sterling Heights, MI, 48310, USA

**Keywords:** Skin cancer, Radiation therapy, Electronic brachytherapy

## Abstract

**Background:**

Squamous cell carcinoma in situ of the digit presents a complex management problem, which is usually treated with surgery or radiation or topical agents. The outcome of the surgical treatment can be an undesirable cosmetic result and loss of function. We report a unique Electronic Brachytherapy technique to treat the digit, which uses a 50 Kv miniaturized X-ray source with specialized applicators.

**Case presentation:**

A 62-year-old African-American male was presented with a 12-month history of gradual darkening of the dorsal-distal middle left finger. Examination revealed a hyper pigmented scaly patch on the proximal to lateral nail fold of the L 3rd finger, nail dystrophy, and vertical split in the lateral section of the nail. The patient underwent evaluation of the lesion by Plastic Surgery with the removal of the lateral nail and a nail bed biopsy. Pathology revealed squamous cell carcinoma in situ with a possible focal positive, deep margin. The patient deliberated over surgical opinions, and eventually decided on radiation. A high dose rate Electronic Brachytherapy system using the XOFT Accent controller delivered dose of 4000 cGy in eight fractions, twice weekly, with at least 48 hours between fractions and treatment prescribed to a depth of 0 to 2 mm. The Xoft unit has specialized skin applicators that permit superficial treatment. Parameters assessed included the efficacy, cosmetic results feasibility, and acute safety of the Electronic Brachytherapy technique.

**Conclusions:**

The patient exhibited moderate redness, hyperpigmentation erythema, desquamation, and Grade 1 to 2 edema acutely (following radiation), which resolved within 1 month of the treatment. Electronic brachytherapy treatment delivery took about 6 minutes, and the total procedure time was about 15 minutes. At the median follow-up of one year, the area revealed excellent cosmesis, and there was no infection or fat necrosis, desquamation, no cancer recurrence, and no evidence of fibrosis at the last follow-up. This suggests that Electronic Brachytherapy was a viable treatment option for this particular patient.

## Background

Skin cancers are a worldwide health problem. In the United States, over two million patients are consistently detected with basal cell or squamous cell carcinoma per year [1]. Squamous cell carcinoma (SCCA-in-Situ) is a cutaneous malignancy, first described by Bowen in the 1900’s. The normal manifestation is an appearance of a slowly growing, sharply defined erythematous, scaly plaque. In darker skin types, it can appear as a hyper pigmented, scaly patch or plaque, with the darker skin type masking the pink color of erythema. It, typically, occurs in the sun exposed areas and in patients over the age of 60, but any age and location can be affected. Eight percent of untreated cases may, eventually, progress to invasive carcinoma. While surgical removal is, typically, the standard treatment, other well-defined treatment options include cryotherapy [2], curettage and electrocautery, surgical excision, laser ablation [3], photodynamic therapy (PDT), topical 5-FU and topical imiquimod [1,4-7]. Conventional radiation is another treatment option. The treatment options include electron beam or photons given daily over a series of 3- 6 weeks. The challenge with conventional radiation techniques is to get an adequate surface dose to the skin, which can be difficult for the extremities and require customized bolus. Some authors have used a water-bath technique, which can improve the surface dose [8]. Other investigators have used a radioactive skin patch to deliver a surface dose to the extremities [9]. High-dose-rate Electronic Brachytherapy has been used to treat skin cancer with the help of the Lepzig surface applicators, and has shown effectiveness with an added advantage of a short radiation treatment using a condensed hypo fractionated regiment (2 times per week). The fractionation scheme used for EBT (500 cgy twice a week for a total dose of 4000 cgy), is identical to that used with High dose rate after-loaded techniques. Electronic Brachytherapy technique (EBT) can be used as a treatment for skin malignancy without the use of a radioactive isotope, but uses a similar hypo fractionated regimen. It is a form of high-dose-rate Electronic Brachytherapy but using a miniaturized x-ray tube, instead of a live isotope. Either surgery or radiation would be a standard treatment approach for squamous cell carcinoma in Situ of the digit [2-4,8].

In general, the dose ranging from 35 to 65 gray with standard fractionation is acceptable. Other fractionation regimens are also acceptable that are not necessarily daily. Optimal external beam radiation treatment regimen, typically, includes fractions that range from 2 to 3.5 centigray. This method ensures greater benefit in terms of better normal tissues preservation and superior aesthetic functional outcome [10].

The risk factors that can predispose individuals to these conditions include prolonged sun exposure, chronic immunosuppressive, and arsenic exposure [11]. The traditional management of Bowen’s disease is surgical excision or Mohs surgery [12]. However, when Bowen’s disease or squamous cell carcinoma occurs in the digits, surgical removal can lead to scar contracture, loss of the fingernail, or amputation in extensive cases. Alternative treatments, such as cryotherapy, photodynamic therapy, and topical creams have other drawbacks, such as variable response rate, dependence on patient compliance for topical creams, and variability in the technique (cryotherapy and PDT8). Others have tried a combination of photodynamic therapy and radiation [13]. There are some additional limited data that suggest carbon dioxide laser vaporization for Bowne’s disease may be effective. All of these nonsurgical and non-radiation approaches are considered in order to spare function but have limitations since they have been only moderately successful [14].

## Case presentation

**Figure 1:**
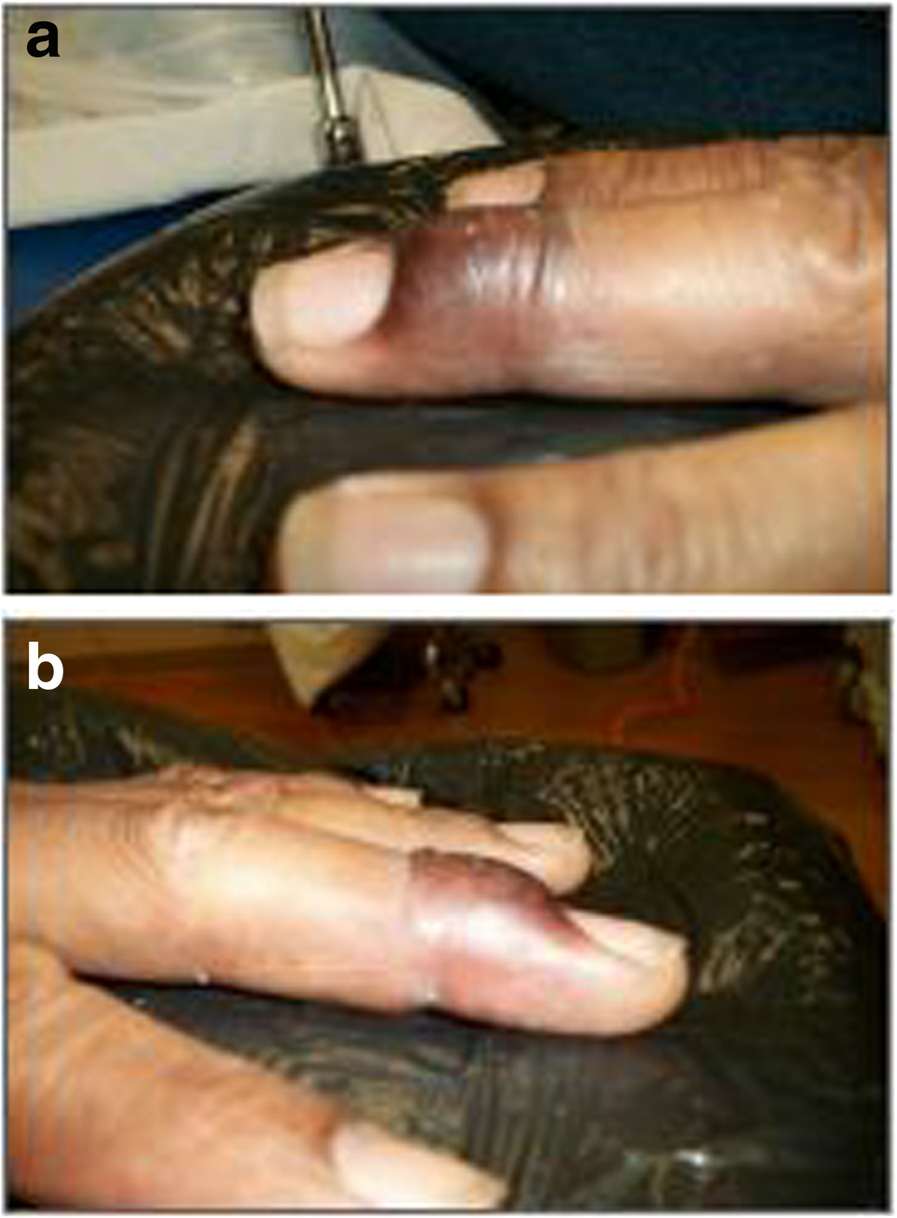
**Pre-treatment appearance of the lesion front and side views.** The Patient presented with a pigmented scaly plaque on the dorsum of the finger and nail bed.

**Figure 2:**
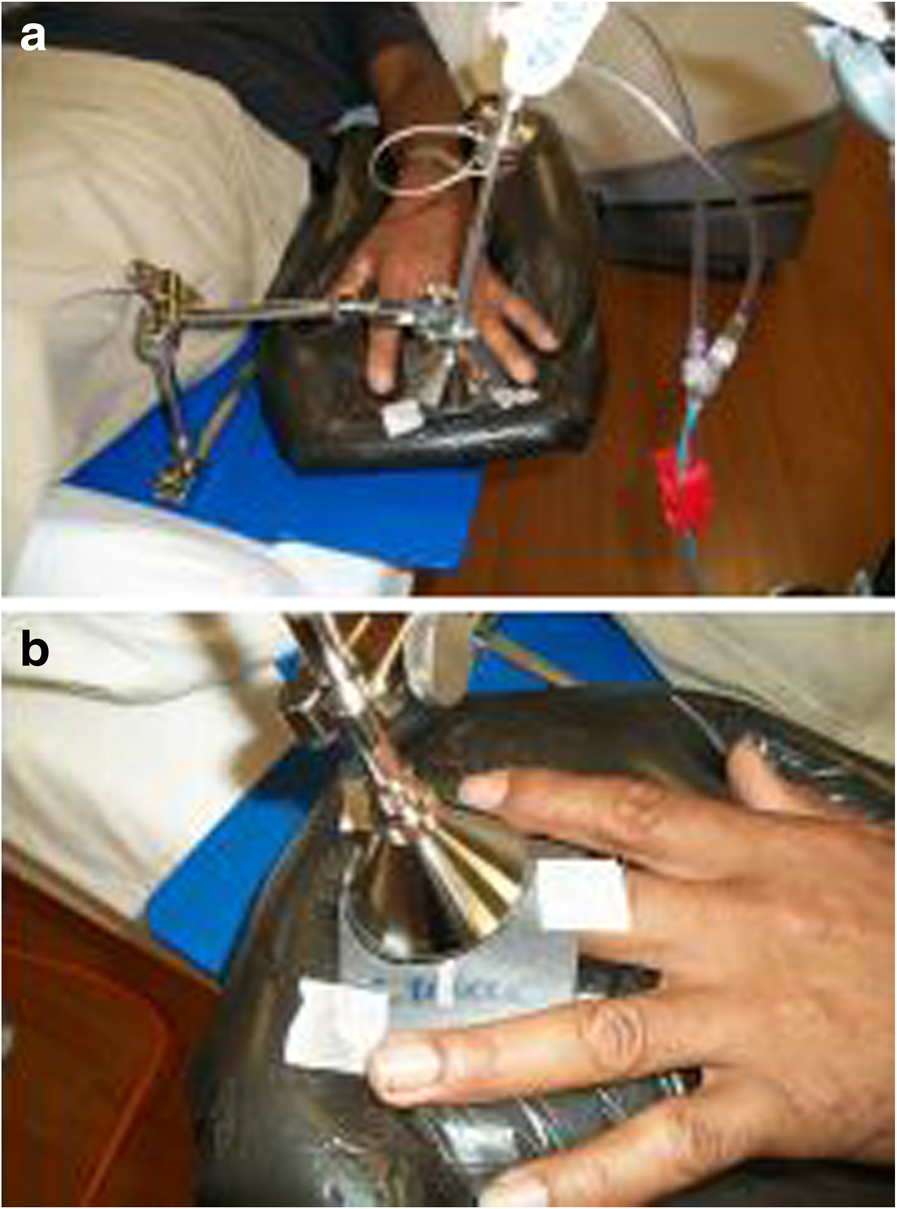
**Clinical set up of treatment with Xoft machine.** Selected treatment cone is placed directly on the surface of the finger. The xoft machine and the electronic source are inserted directly into the hollow cone to allow the dose delivery directly to the area.

**Figure 3:**
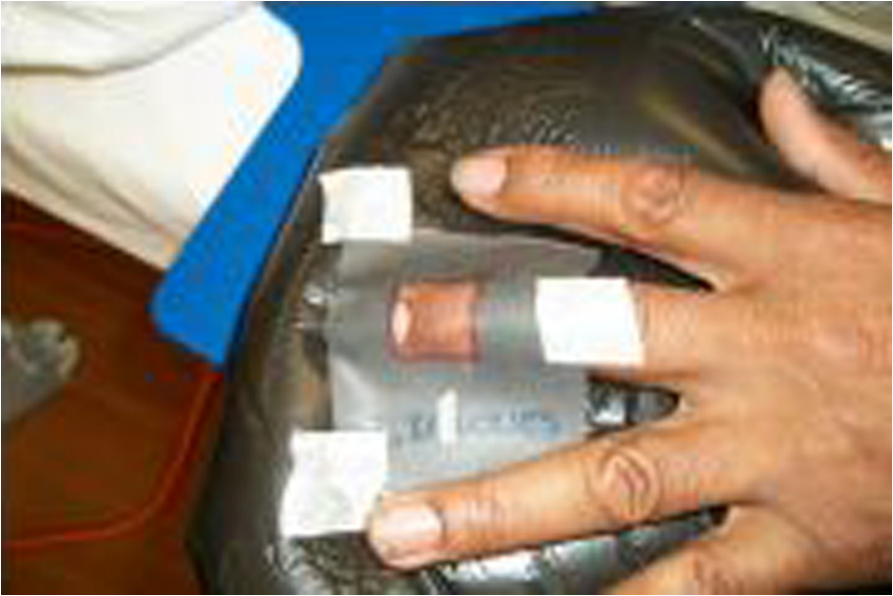
**Customized lead cut out used on the digit to protect uninvolved areas.** The half-value of lead is calculated to shield 100% of non-involved tissues. The cut out is designed based on the clinical shape and size of the lesion with a small margin.

**Figure 4:**
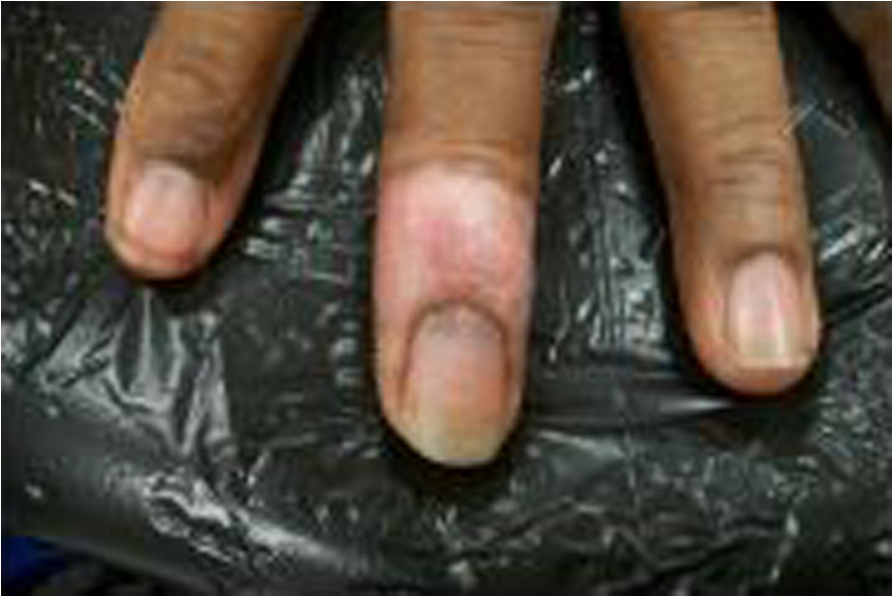
**Post-treatment, 6 month, hypopigmentation was seen in areas of re-pigmentation.** The patient has complete and normal function and good cosmesis.

In this case study, the patient was, primarily, treated with a superficial Electronic Brachytherapy technique using the surface applicators. Customized lead shield was used to minimize the dose to the unintended areas (Figures 1, 2, 3, 4).

The 62-year-old African-American male presented the condition of squamous cell carcinoma in Situ of the digit. Treatment planning and simulation was performed using customized mobilization and thermoplastic mold around the hand and fingers and the patient was lying supine on the CT simulator couch. This allowed complete immobilization of the fingers as well as the hand. CT scans of the extremity were taken to evaluate the lesion depth for treatment, planning, and the appropriate surface areas for the custom applicator. Acceptable clinical target volume around the tumor was a 3 mm margin due to the sharp fall off of the source.

Then, a customized lead shield (used as a cut out) and a 3.5 cm cone applicator were selected. The half-value layer of the lead was calculated in order to block the unintended areas and decrease the amount of dose to the uninvolved tissues. After simulation, the depth calculation was based on the thickness of the lesion, and then, the treatment was delivered to the patient. The Xoft skin applicators are calibrated with fixed depth dose curves. For example if you prescribe to .5 cm from the applicator surface, your surface dose is 1000 cGy. The comprehensive calibration processes ensured correct dose output of these applicators prior to each treatment. Primary observations revealed the average normal dose-rate output of the skin surface for the 35 mm applicator was 1.35 gray per minute with a positive/negative 5% variation. The output variation is within 2%. Calculations of the effective source-surface distance were carried out on the air-gap measurements for all applicator sizes, and the field flatness and symmetry were well within 5%. Calibration in this case study was identical to cases reported elsewhere on quality assurance for this technique [15].

Clinically, the patient had a fair amount of keratotic epithelialization, and thus, the initial treatment prescription was to 2 mm in depth. After the first three fractions, the patient developed severe irritation and the lesion appeared to flatten, and thus, the doses for the remaining fractions delivered at the skin-surface. The patient received a total dose of 4000 centigray delivered in 500 centigray fractions over eight fractions twice a week with 48 hours between fractions. The total time for radiation delivery was about 5 minutes, and the total mean procedure time was about 15 minutes from the start to finish (including the clinical set up). The patient tolerated the treatment well and had no recurrence of the cancer in the one-year follow-up and overall cosmesis was excellent in the patient. There was no infection, fat necrosis, desquamation, or cancer recurrences. There was no evidence of fibrosis, at the last follow-up session. There has been no recurrence of tumor. At the site of the radiation application, mild skin desquamation developed in the weeks following the treatment. This reaction healed with minimal fibrosis and epithelialized quickly. There was no adverse reaction to the radiation, except for the appearance of a small 2 mm area of moist desquamation, appropriately one week after the last treatment, which resolved with topical agents. The patient also experienced hypopigmentation that is slowly resolving.

## Discussion

Ever since its introduction in 2009, the Xoft Accent Electronic Brachytherapy unit with skin applicators has been used to treat skin cancer. The Xoft miniature x-ray tube generates 50 kV photon radiations, which requires minimal shielding. The cone-shaped skin applicators come in a variety of sizes from 5 mm – 50 mm, which allows precise placement of the radiation directly to the lesion. The patient can be treated in any position, and in almost any location with minimal shielding. Electronic Brachytherapy is a unique cancer treatment modality, which is an alternative for the treatment of the digit, when compared with external beam radiation therapy, chemotherapy and topical agents. One of the advantages of Electronic Brachytherapy is that the treatment is relatively sophisticated, much localized, non-invasive, and allows superficial delivery of radiation in a variety of settings. Because of the twice-a-week treatment and only a total of 8 fractions, this technique has a higher degree of patient acceptance and compliance. It may be suitable for lesions in an unusual location, the elderly, and in cases when patients are unable to tolerate five weeks of daily treatment. The significant advantage is patient convenience and the lack of regulatory issue associated with an after-loaded source. This technique requires less physicist supervision and the machine can be operated by the therapists, increasing treatment efficiency. There is limited data about the long term effectiveness of this technique and the largest experience has short follow up and excellent tumor control similar to what is seen with external beam therapy (16) Although, we did see acute forms of skin reaction, such as erythema, dry desquamation, and mild moist desquamation, these conditions healed quickly with minimal toxicity, and are due to the high surface dose in this technique. Hyperpigmentation or hypopigmentation of radiated skin may develop as chronic reaction within a couple of months after completion of the treatment, but this reaction will fade. It may also progress to a vitiligo appearance in some patients [16].

## Conclusion

Superficial skin tumors of the digit are a challenging management problem, and can be effectively treated by Electronic Brachytherapy (EBT) using the superficial skin applicators. EBT to the skin, for squamous cell carcinoma in situ, is a feasible alternative to surgery for squamous cell carcinoma of the digit. Further exploration and more extensive follow-up on this method will help to gain better insights and shed light in the direction of clinical usefulness of the procedure. This case study positively indicates that Electronic Brachytherapy using superficial skin applicators is a local therapeutic solution for the treatment of this case. It is a convenient option and a non-invasive therapy with satisfactory cosmetic results and outcome for this patient. The convenience and portability of this technique can allow more patients access to care.

### Consent

Written informed consent was obtained from the patient for publication of this case report and accompanying images. A copy of the written consent is available for review by the Editor-in-Chief of this journal and can be sent by fax or electronically for review.

## Competing interests

The authors declare that they have no competing interests and received no financial compensation for this manuscript.

## Authors’ contributions

VEA was the major contributor to case selection, analysis, development of the manuscript deciding treatment guidelines, and clinical delivery. She also has written, proofread and edited this submission. AW provided assistance on the proofreading and formatting of the paper as well as content review. She has read and edited this submission. Both authors have read and approved the final manuscript.

## Authors’ information

VEA is a radiation oncologist with an interest in Electronic Brachytherapy and emerging technologies with radiation techniques. She has a Master’s degree in Health Services Administration from the University of Michigan as well as MD from that institution. VEA is interested in novel treatments that produce excellent outcome with improved cost benefit and quality of life for patients.
